# Supplementary material for: Van der Waals lattice-induced colossal magnetoresistance in Cr2Ge2Te6 thin flakes
Source: Nat Commun. 2022 Oct 28;13:6428. doi: 10.1038/s41467-022-34193-w (PMC9616818; doi:10.1038/s41467-022-34193-w)
Supplement: Supplementary file 1 — Supplementary Information [file 41467_2022_34193_MOESM1_ESM.pdf]

Supplementary Information for

"Van der Waals lattice-induced colossal magnetoresistance effect in $Cr_2Ge_2Te_6$ thin flakes"

Wenxuan Zhu, Cheng Song,* Lei Han, Tingwen Guo, Hua Bai, Feng Pan*

Key Laboratory of Advanced Materials, School of Materials Science and Engineering, Beijing Innovation Center for Future Chips, Tsinghua University, Beijing 100084, China.

*E-mail: songcheng@mail.tsinghua.edu.cn, panf@mail.tsinghua.edu.cn

Supplementary Note 1. Carrier type of CGT

The carrier type of CGT is measured through the field effect curve and Hall effect, which both illustrates the hole-type carrier of CGT. Supplementary Fig. 1a schematically shows the fabricated CGT field effect transistor (FET). The measured field effect curves shown in Supplementary Fig. 1b exhibits the feature of p-type FET, reflecting the hole-carrier in CGT. Both cyclicity (20 cycles) and leakage current are also shown. This is furtherly supported by the measurement of Hall effect in Supplementary Fig. 1d, whose setup is shown in Supplementary Fig. 1c.

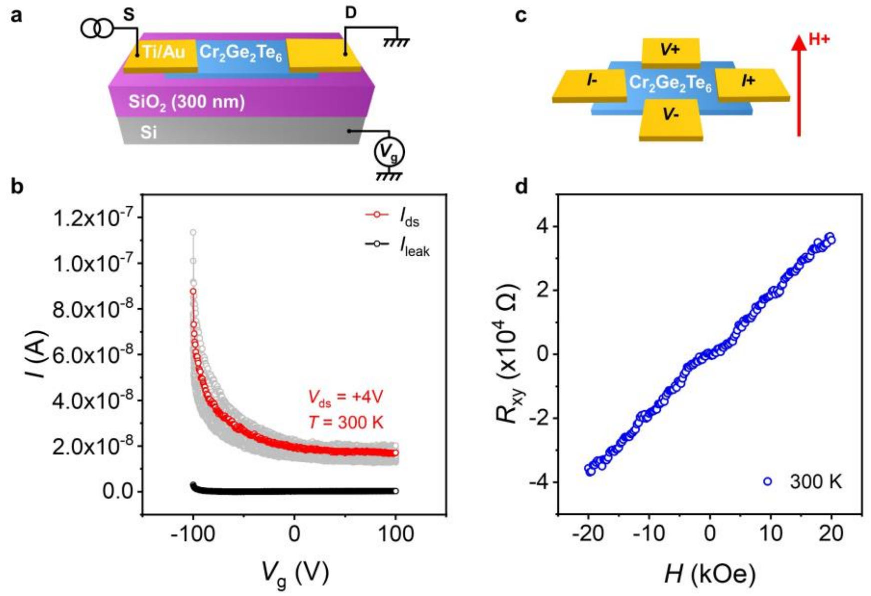

Supplementary Figure 1. a, Schematic of CGT field effect transistor. **b,** Field effect curves measured at 300 K. The cyclicity (20 cycles) of source-drain current (I_{ds}) and leakage current (I_{leak}) are exhibited in grey and black, respectively. One typical curve is highlighted in red. **c,** Measurement setup of Hall effect. **d,** Field-dependent Hall resistance (R_{xy}) of CGT measured at 300 K.

Supplementary Note 2. Field scan of R_{nl}

Supplementary Fig. 2 shows the result of the field-dependent R_{nl}. With the applied magnetic field of 5 T, the saturation of R_{nl} indicates the fully alignment of the magnetization by the magnetic field. The larger saturation filed with more significant resistance change along in-plane direction (**x**, **y**) compared to the out-of-plane direction (**z**) shown in the inset also reflects the magnetic easy axis is close to the out-of-plane direction.

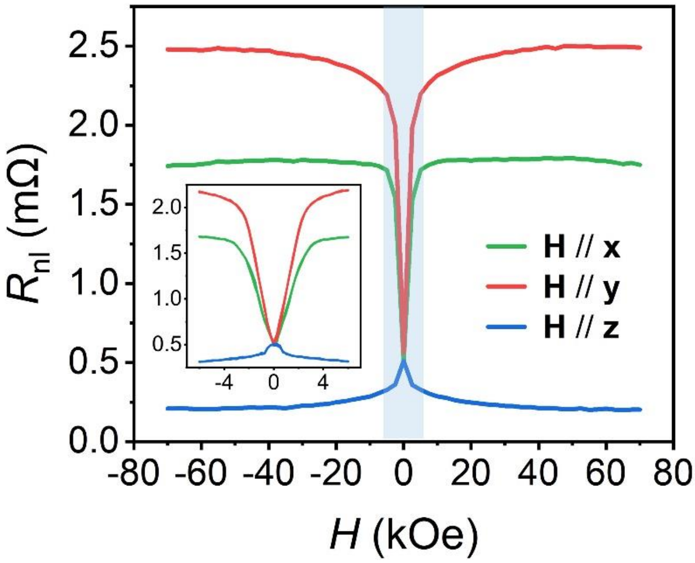

Supplementary Figure 2. Field-dependent nonlocal resistance (R_{nl}) with the magnetic field different directions. The inset highlights the results around zero field.

Supplementary Note 3. Angular nonlocal MR with tungsten detector

The control experiment with the injector of platinum and detector of tungsten was perform. The schematic of the measurement is shown in Supplementary Fig. 3a. Due to the opposite spin Hall angle between Pt and W, in the spin-related transport, the polarity of the signal will be inversed with W detector (Pt inject, W detect) compared to Pt (Pt inject, Pt detect). In contrast, as illustrated in Supplementary Fig. 3b, the results with W detector is similar to that of Pt shown in Fig. 3c in the main text. The high R_{nl} is obtained with **M** // **y** and **M** // **x** in β and γ scan, respectively. With **M** // **z**, it shows the low R_{nl}. Therefore, the spin-related transport is negligible in our nonlocal devices.

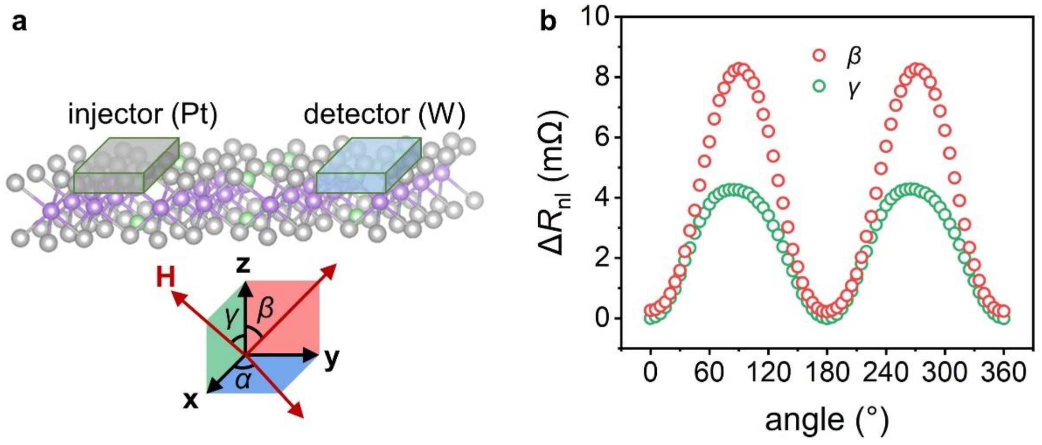

Supplementary Figure 3. a, Schematic of the measurement with the definition of azimuthal angles and directions. **b,** Angle-dependent amplitude of nonlocal resistance ΔR_{nl} ($R_{nl} - R_{nl}^{z}$) of the β and γ scans with the 5 T magnetic field at 10 K.

Supplementary Note 4. Intralayer lattice induced modulation

We fabricated the devices along different directions on the same sample to further verify the in-plane anisotropy induced deviations observed in Fig. 5 in the main text, as shown by the optical image in Supplementary Fig. 4a. The crystal orientation of the sample was determined by the EBSD and the direction in green was calibrated as $[8\ \overline{17}\ 9\ 0]$ as highlighted in Supplementary Fig. 4b. The direction of the electrode and the direction in green is defined as θ and Supplementary Fig. 4c and d shows the angular R_{nl} in α scan and its derivative of the devices with θ = 0 °, 60 °, 90 °, respectively. The deviations in devices with θ = 0 ° and 60 ° is similar and opposite to the device with θ = 90 °, which is consistent with the period of crystal directions and the principle proposed in the main text. The derivative can also reflect the opposite behavior of deviation. As shown in Supplementary Fig. 4d, the deviations of α scans with opposite deviations are not smooth with opposite trend around 180 °. The derivatives of the R_{nl} signal shown in Fig. 5b in the main text also exhibit similar features in Supplementary Fig. 4e. The deviation can also be observed in the measurement of local MR as illustrated in Supplementary Fig. 4f.

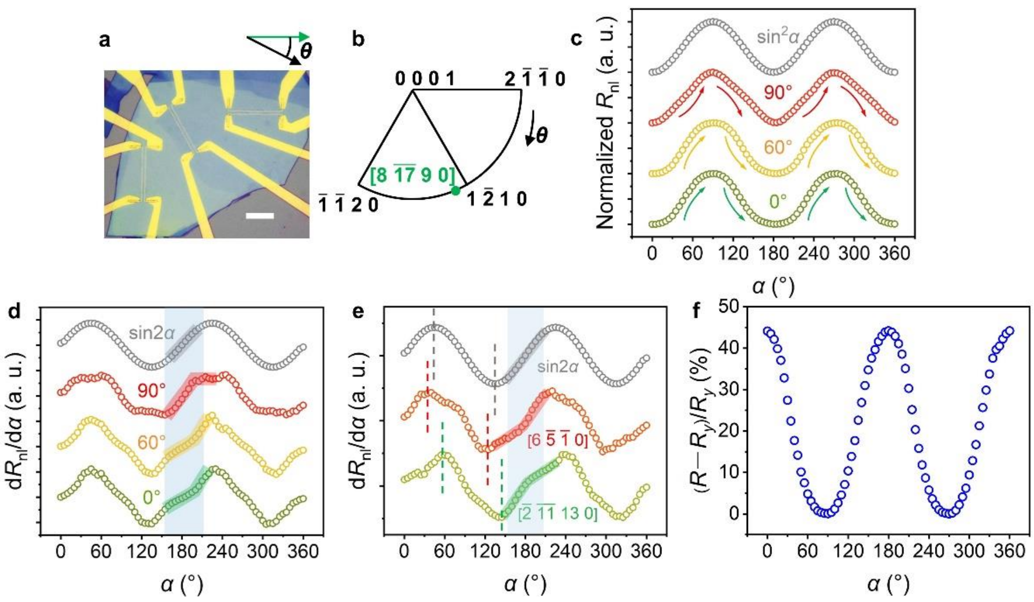

Supplementary Figure 4. a, Optical microscope image of the devices on the same sample along different directions. Scale bar: 10 μm. **b,** Schematic of the crystal directions of the sample. The direction in red is highlighted by the green point. **c,d,** Normalized R_{nl} (**c**) and the derivatives (**d**) in α scan of the devices along different directions. The angle between the direction of the electrode and the direction in green is defined as θ. **e,** Derivatives of the R_{nl} signal of Fig. 5b in the main text. **f,** Local angular MR in α scan.

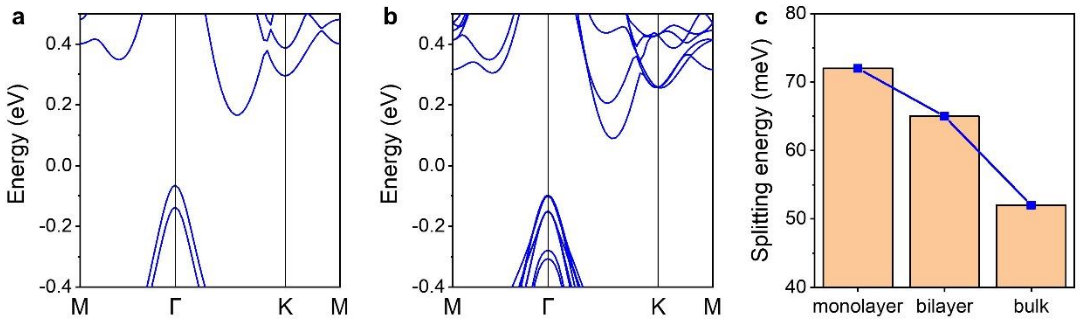

Supplementary Figure 5. **a**,**b** Calculated band structure of monolayer (a) and bulk (b) CGT with the out-of-plane magnetization. **c**, Comparison of splitting energy among monolayer, bilayer and bulk CGT.

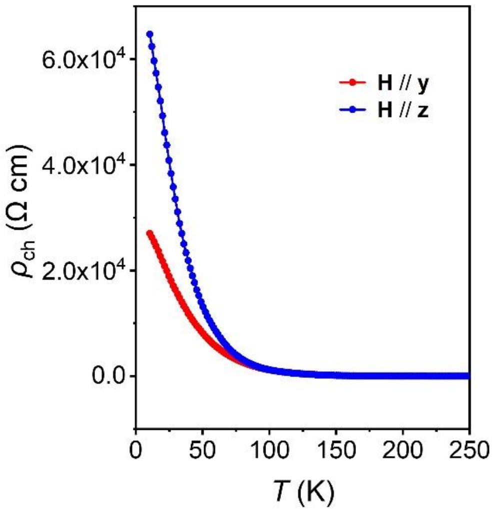

Supplementary Figure 6. Temperature-dependent channel resistivity (ρ_{ch}) with magnetization along in-plane (**y**) and out-of-plane (**z**) directions under 5 T applied magnetic field.

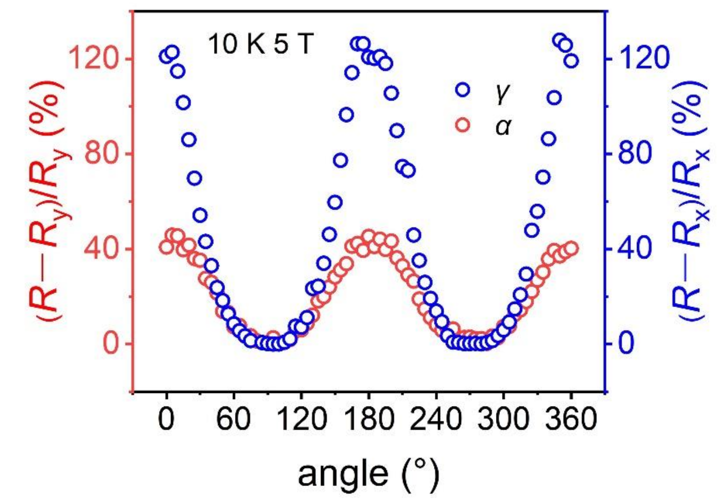

Supplementary Figure 7. Local angular MR effect in γ and α scans.

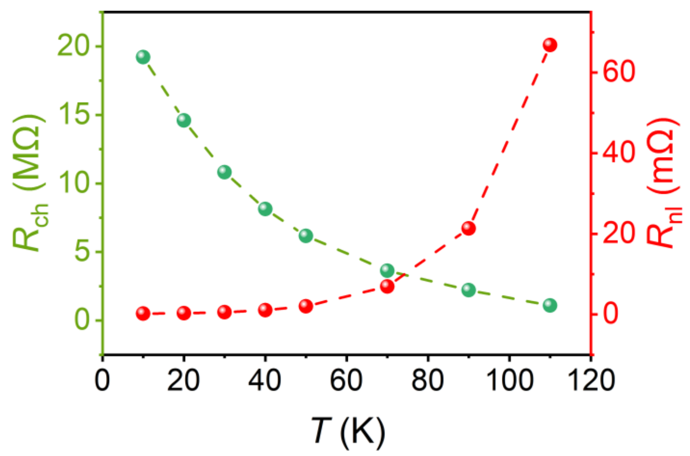

Supplementary Figure 8. Temperature-dependent channel resistance R_{ch} (green) and nonlocal resistance R_{nl} (red).

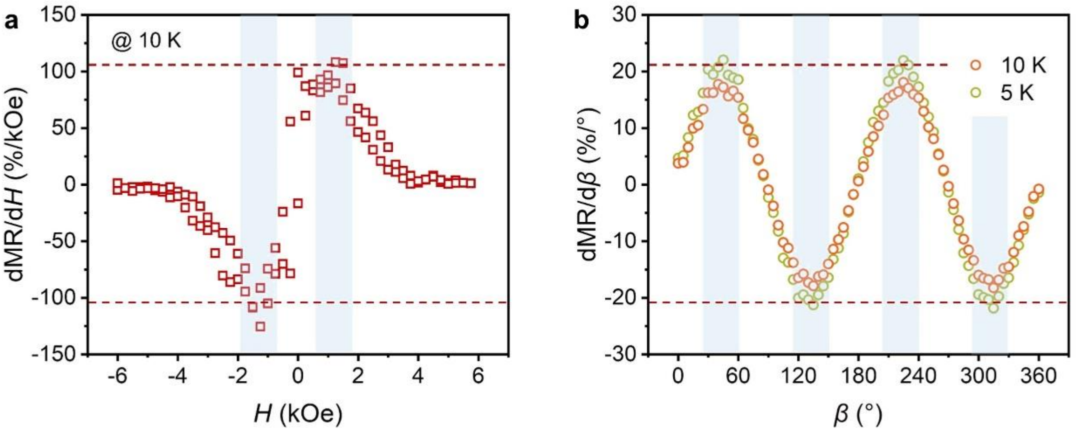

Supplementary Figure 9. Sensitivity of the nonlocal device to the magnitude and direction of the external magnetic field. Derivatives of nonlocal MR in the field scan (a) and β scan (b). The peaks are highlighted in blue.

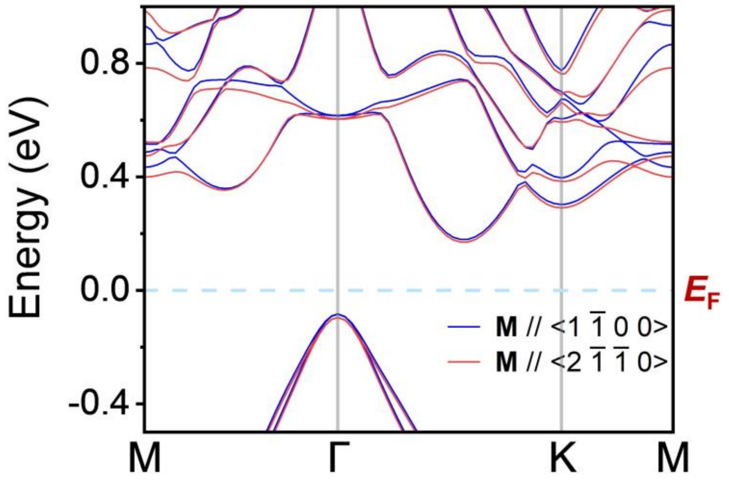

Supplementary Figure 10. Band structures with magnetization along in-plane directions. The fermi level is set to zero. The band crossing with **M** // <1$\bar{1}$00> becomes gapped with **M** // <2$\bar{1}\bar{1}$0>, which is circled in black.
